# Supplementary material for: Bus bunching as a synchronisation phenomenon
Source: Sci Rep. 2019 May 3;9:6887. doi: 10.1038/s41598-019-43310-7 (PMC6499774; doi:10.1038/s41598-019-43310-7)
Supplement: Supplementary file 1 — Supplementary information [file 41598_2019_43310_MOESM1_ESM.pdf]

Bus bunching as a synchronisation phenomenon: Supplementary information

Vee-Liem Saw,[1,2] Ning Ning Chung,[3] Wei Liang Quek,[1] Yi En Ian Pang,[1] and Lock Yue Chew[1,2,3,*]

[1]*Division of Physics and Applied Physics,*
School of Physical and Mathematical Sciences, 21 Nanyang Link,
Nanyang Technological University, Singapore 637371

[2]*Data Science and Artificial Intelligence Research Centre,*
Block N4 #02a-32, Nanyang Avenue,
Nanyang Technological University, Singapore 639798

[3]*Complexity Institute, 61 Nanyang Drive,*
Nanyang Technological University, Singapore 637335

(Dated: April 9, 2019)

* lockyue@ntu.edu.sg

I. ANALYTICAL DERIVATION OF THE PHASE TRANSITION TO COMPLETE PHASE LOCKING OF ALL N BUSES SERVING M STAGGERED BUS STOPS IN A LOOP

Consider $N = 2$ buses with natural angular frequencies $\omega_1 > \omega_2$ ($\omega_i = 2\pi f_i = 2\pi/T_i$) serving $M = 1$ bus stop in a loop. Suppose that the coupling $k := s/l$ is strong enough such that these two buses are phase locked. (Recall that s and l are the people arrival and loading rates, respectively.) In that case, these two buses would always bunch at the bus stop and share the loading of people. Once everybody has been picked up, the two buses leave together, with the faster one pulling away. After one revolution, the faster one returns to the bus stop and begins picking up people. But before finishing, the slower one arrives (because k is strong enough such that there are many people waiting at the bus stop) and the two buses share loading. These two buses are in such an equilibrium which repeats over and over.

In Fig. 1, (a) is the moment when the two buses just leave the bus stop after picking up everybody, (b) is when the fast bus just arrives after one revolution, (c) is when the slow bus just arrives, and (d) is when both buses have finished picking up everybody and leave. The time elapsed from (a) to (b) is T_1, from (b) to (c) is $T_2 - T_1$, from (c) to (d) is τ_{shared}, where τ_{shared} is the duration when these two buses share loading. The total number of people to be picked up is s times the total time elapsed from (a) to (d), which is $T_2 + \tau_{\text{shared}}$. These people are picked up by:

1. Only the fast bus $= l(T_2 - T_1)$.

2. Shared by the fast and slow buses $= 2l\tau_{\text{shared}}$.

The critical transition between no phase locking and phase locking is when $\tau_{\text{shared}} = 0$. In that case,

$$sT_2 = l(T_2 - T_1) \tag{1}$$

$$k_c := \frac{s}{l} \tag{2}$$

$$= 1 - \frac{T_1}{T_2} \tag{3}$$

$$= 1 - \frac{f_2}{f_1} \tag{4}$$

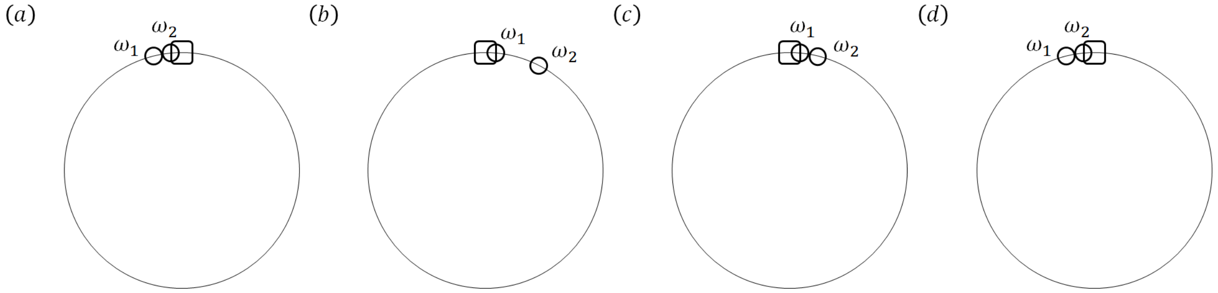

FIG. 1. $N = 2$ buses serving $M = 1$ bus stop in a loop, where k is strong enough such that they are phase locked.

$$= 1 - \frac{\omega_2}{\omega_1}. \tag{5}$$

If there are M staggered bus stops, then these sequence of events repeat at every bus stop. At the critical transition, $\tau_{\text{shared}} = 0$ and the total number of people to pick up is still sT_2. However, the time interval for the fast bus to pick up all people is only $(T_2 - T_1)/M$ since there are M staggered bus stops in one revolution. Then, we have

$$sT_2 = \frac{l(T_2 - T_1)}{M} \tag{6}$$

$$k_c = \frac{1}{M}\left(1 - \frac{T_1}{T_2}\right) \tag{7}$$

$$= \frac{1}{M}\left(1 - \frac{f_2}{f_1}\right) \tag{8}$$

$$= \frac{1}{M}\left(1 - \frac{\omega_2}{\omega_1}\right). \tag{9}$$

So with M bus stops, each bus stop multiplies the coupling strength. Hence, only one M-th of the coupling strength with one bus stop is required when there are M bus stops.

Let us now consider N buses with angular frequencies $\omega_1 > \cdots > \omega_N$ serving $M = 1$ bus stop in a loop, and we know that having M staggered bus stops would be one M-th of k_c for $M = 1$. The total number of people to pick up is $s(T_N + \tau_{\text{shared}})$, since all buses have to wait for the slowest bus to reach the bus stop, and then all buses would share the load over the duration τ_{shared}. These people are picked up by:

1. Only the first bus $= l(T_2 - T_1)$.

2. Shared by only the first and second buses $= 2l(T_3 - T_2)$.

3. Shared by only the first, second and third buses $= 3l(T_4 - T_3)$.

4. $\cdots$

5. Shared by only the first $N-1$ buses $= (N-1)l(T_N - T_{N-1})$.

6. Shared by all buses $= Nl\tau_{\text{shared}}$.

The critical transition between complete and partial phase locking is when $\tau_{\text{shared}} = 0$. In that case,

$$sT_N = l\left[T_2 - T_1 + 2T_3 - 2T_2 + 3T_4 - 3T_3 + \cdots + (N-1)T_N - (N-1)T_{N-1}\right] \tag{10}$$

$$= l\left[(N-1)T_N - \sum_{i=1}^{N-1} T_i\right] \tag{11}$$

$$k_c = \sum_{i=1}^{N-1}\left(1 - \frac{T_i}{T_N}\right). \tag{12}$$

Thus, the critical transition between complete and partial phase locking for the general case of N buses serving M staggered bus stops in a loop is:

$$k_c = \frac{1}{M}\sum_{i=1}^{N-1}\left(1 - \frac{\omega_N}{\omega_i}\right), \tag{13}$$

where $\omega_N/\omega_i = f_N/f_i = T_i/T_N$.
